# Modeling of Blood–Brain Barrier (BBB) Dysfunction and Immune Cell Migration Using Human BBB-on-a-Chip for Drug Discovery Research

**DOI:** 10.3390/ijms25126496

**Published:** 2024-06-12

**Authors:** Masato Ohbuchi, Mayu Shibuta, Kazuhiro Tetsuka, Haruna Sasaki-Iwaoka, Masayo Oishi, Fumitaka Shimizu, Yasuhisa Nagasaka

**Affiliations:** 1 Applied Research & Operations, Astellas Pharma Inc., Tsukuba 305-8585, Ibaraki, Japan; mayu.shibuta@astellas.com (M.S.); kazuhiro.tetsuka@astellas.com (K.T.); haruna.iwaoka@astellas.com (H.S.-I.); masayo.oishi@astellas.com (M.O.); yasuhisa.nagasaka@astellas.com (Y.N.); 2 Department of Neurology and Clinical Neuroscience, Graduate School of Medicine, Yamaguchi University, Ube 755-8505, Yamaguchi, Japan; fshimizu@yamaguchi-u.ac.jp

**Keywords:** BBB-on-a-chip, barrier dysfunction, immune cell migration, T cell, drug discovery research

## Abstract

Blood–brain barrier (BBB) dysfunction is a key feature in neuroimmunological and neurodegenerative diseases. In this study, we developed a microfluidic human BBB-on-a-chip to model barrier dysfunction and immune cell migration using immortalized TY10 brain endothelial cells, pericytes, and astrocytes. It was found that immortalized TY10 brain endothelial cells developed a microvascular structure under flow. Pericytes were localized on the basal side surrounding the TY10 microvascular structure, showing an in vivo-like structure. Barrier integrity increased under co-culture with pericytes. In addition, both ethylenediaminetetraacetic acid (EDTA) and anti-Claudin-5 (CLDN5) neutralizing antibody caused a decrease in the transendothelial electrical resistance (TEER). EDTA caused the leakage of 20 kDa dextran, suggesting different effects on the BBB based on the mechanism of action, whereas anti-CLDN5 antibody did not cause leakage. In the tri-culture model, human T cells migrated through endothelial vessels towards basal C-X-C motif chemokine ligand 12 (CXCL12). The live-imaging analysis confirmed the extravasation of fluorescence-labelled T cells in a CXCL12-concentration- and time-dependent manner. Our BBB model had an in vivo-like structure and successfully represented barrier dysfunction and transendothelial T cell migration. In addition, our study suggests that the inhibition of CLDN5 attenuates the BBB in humans. This platform has various potential uses in relation to the BBB in both drug discovery research and in elucidating the mechanisms of central nervous system diseases.

## 1. Introduction

The blood–brain barrier (BBB) has a crucial structural and functional role in restricting the passage of soluble mediators and leukocytes from the blood into the central nervous system (CNS) [1]. The BBB is a neurovascular unit formed of microvascular endothelial cells, pericytes, perivascular astrocytes, and neurons [2]. In addition, BBB dysfunction is a key feature of several neurological and neuroimmunological diseases [3]. Although studies using experimental animal models and post-mortem brains provide valuable evidence, functional and detailed mechanistic investigation remains difficult. Thus, establishing an in vitro human BBB model would allow investigation using functional assays. However, the modeling of BBB dysfunction using in vitro human systems is currently limited.

To represent the BBB in vitro, the Transwell model is widely used because of its cost effectiveness and simplicity [4,5]. However, this platform has certain limitations: (1) a lack of shear stress on endothelial cell differentiation; (2) limited direct cell-to-cell interaction between brain endothelia and pericytes due to the presence of the insert membrane; and (3) difficulty in real-time imaging of immune cell migration. In particular, shear stress is important for BBB barrier function and transporter expression [6,7]. Fluid flow is also crucial in modeling leukocyte–endothelial interactions and transmigration [8,9]. 

A wide variety of microfluidic-based BBB models have been proposed for different applications. BBB-on-a-chip models have been utilized to investigate the brain penetration of small compounds [10,11], antibodies [11,12], and nanoparticles [11,13,14]. Various studies have also performed toxicological evaluations of the neurovascular unit [15,16,17]. Moreover, neuroinflammation [18,19,20,21] and neuroinfection (e.g., SARS-CoV-2 and *Cryptococcus neoformans*) models [22,23] have been designed to better understand the BBB’s pathology and infection mechanism. Using a Parkinson’s disease model, Pediaditakis et al. explored the alpha-Synuclein (aSyn) pathology by exposing a human brain-chip containing BBB components and hiSPC-derived neurons to aSyn fibrils [24]. In addition, Vatine et al. developed a BBB-on-a-chip for personalized medicine using hiPSC-derived BMECs and neural progenitor cells from individuals with Huntington’s disease (HD) [20]. The results showed higher permeability in the HD group than the healthy control group and revealed inter-individual differences in the BBB phenotype [20].

BBB-on-a-chip models have seen great developments in the last decade. Nevertheless, there is still no widely accepted BBB-on-a-chip model for drug discovery research. Several challenges limit the applicability of these models in pharmaceutical studies: (1) Throughput: drug testing typically requires the use of multiple well plates (e.g., 24 wells or more), but most current models do not have this capability. (2) Device material: most BBB-on-a-chip models are fabricated using polydimethylsiloxane (PDMS), which is known to adsorb small hydrophobic molecules [25]. This makes drug testing more difficult, especially for lipophilic drugs. (3) The development of a transendothelial electrical resistance (TEER) sensor: the widely used chopstick electrode is too large to be integrated into a microchip. Thus, the development of a novel sensor or instrument for BBB-on-a-chip models that can measure TEER with a high throughput is required. (4) Improved BBB properties: appropriate cell source selection is a common problem. For example, primary brain endothelial cells can rapidly lose their characteristics after isolation due to dedifferentiation. They can also exhibit donor-to-donor variability. Human iPSC-derived brain endothelial cells (hiPS-BEC) exhibit strong epithelial-like gene expression and phenotype [20,26]. Using the well-known human brain endothelial cell line hCMEC/D3 is limited by its low barrier integrity [27]. In addition, hCMEC/d3 has relatively low expression of Claudin-5 (CLDN5), an important tight junction protein in the BBB [28]. The BBB component cells, other than brain microvascular endothelial cells, such as pericytes and astrocytes, should also be included to improve BBB function [19,23,29]. (5) The further characterization of BBB-on-a-chip models: barrier integrity in BBB-on-a-chip models has been evaluated using general barrier disrupting agents (e.g., ethylenediaminetetraacetic acid (EDTA)), toxins (e.g., lipopolysaccharide), and inflammatory cytokines (e.g., TNFα or IL-1β) [18,19,20,21]. However, these compounds affect both brain capillary endothelial cells and also epithelial barriers and peripheral blood vessels. To assess whether an established BBB model reflects the characteristics of the brain capillary endothelium, further characterization using compounds selective for BBB-specific molecules (e.g., anti-CLDN5) is necessary.

The OrganoPlate platform can utilize 40 or more chips at a time and does not contain PDMS. TY10 is a conditionally immortalized human brain microvascular endothelial cell line that exhibits leak-tight barrier function with comparable CLDN5 expression to primary human brain endothelial cells [30,31]. Wevers et al. reported the construction of a TY10 Organoplate model to assess a BBB-penetrating antibody [12]. To date, however, no study has reported the use of TY10 in a microfluidic device to model BBB dysfunction. Further, the characterization of the Organoplate model’s barrier integrity using TEER measurements is lacking. In addition, pericytes and astrocytes are co-cultured at a distance from endothelial cells, and the effect of co-culture with pericytes on barrier function has not been investigated. Various studies used the OrganoPlate model with hiPS-BEC to investigate drug delivery to the CNS [10,32], but these did not model BBB disruption. Nair et al. reported an OrganoPlate model of immune cell migration using primary brain endothelial cells [21]. However, this model did not include pericytes and astrocytes. Given the routine and repeated use of BBB-on-a-chip models in drug discovery research, developing a model using a scalable cell source is desirable. 

In the present study, we developed a novel BBB-on-a-chip to model BBB dysfunction and immune cell migration for drug discovery research utilizing the OrganoPlate platform. In this model, immortalized human pericytes and astrocytes were proximally co-cultured in the middle extracellular matrix (ECM) close to TY10 endothelia. The model mimics the structure of the BBB in vivo, to allow direct cell-to-cell interaction. The effect of co-culturing pericytes on the TY10 microfluidic model was characterized using a dextran assay and TEER measurements. In addition to EDTA, we further characterized this model using anti-CLDN5, a selective antibody that targets a BBB-specific molecule. Finally, we performed real-time imaging to model transendothelial immune cell migration. 

## 2. Results

### 2.1. Microfluidic Culture of Human Brain Endothelial Cell (TY10)

**Figure 1:**
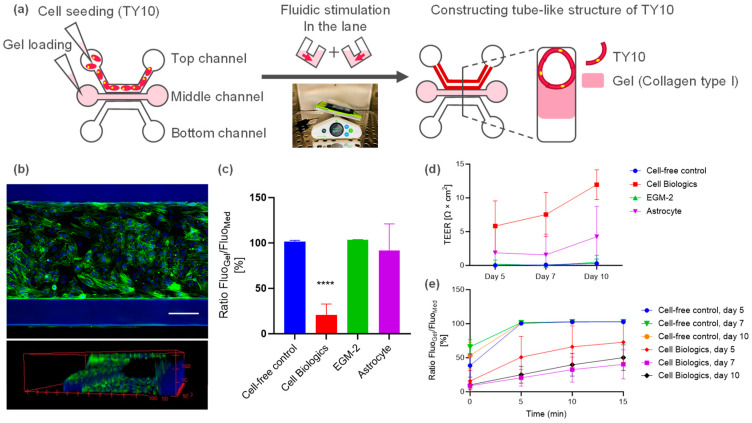
Microfluidic TY10 human brain endothelial model using OrganoPlate. (**a**) Schematic representation of the constructed BBB model. Collagen type I ECM gel was seeded in the middle channel and TY10 was seeded in the top channel after collagen gelation. In the culture with perfusion stimulation, a TY10 microvessel grew against the ECM gel and a tube-like structure was constructed. (**b**) Confocal image (top) and reconstruction of a confocal z-stack image (bottom) of a TY10 monoculture microvessel stained with ActinGreen (green) and Hoechst33342 (blue) on day 10. Scale bar is 100 µm. (**c**) Dextran leakage on day 10 in various commercially available cell culture media, namely, Cell Biologics endothelium medium (Cell Biologics), Lonza EGM-2 medium (EGM-2), and ScienCell Astrocyte medium (Astrocyte). Fluorescent images for quantification were acquired 5 min after adding dextran. Data were analyzed using ordinary one-way ANOVA Dunnett’s multiple comparison tests (**** *p* < 0.0001). (**d**) TEER values of the TY10 microvessel at different time points. (**e**) Dextran leakage in the chip at different time points for the cell-free control and TY10 monoculture microvessel.

TY10 cells were cultured using OrganoPlate 3-lane to develop a microfluidic model of human brain endothelia (Figure 1a). TY10 cells were proliferated in the top lane. On day 10 after the initiation of the fluidic culture, a vessel-like tubule structure of TY10 was confirmed via nuclear and actin staining (Figure 1b). 

Barrier function was evaluated using the dextran leakage assay and TEER measurements. Initially, TY10 microvessels were cultured using various commercially available cell culture media, namely, Cell Biologics (CB), EGM-2, and astrocyte medium (AM). The CB medium significantly prevented dextran leakage (Figure 1c) and showed detectable TEER values of approx. 10 Ω·cm^2^ on day 10 (Figure 1d). In addition, the CB medium significantly prevented dextran leakage from day 7 to day 10 (Figure 1e). These results indicated the leak-tight barrier function of the TY10 microvessels. The AM exhibited detectable TEER values (Figure 1d). When TY10 cells were cultured using EGM-2, the barrier was very leaky, and no detectable TEER was observed (Figure 1c,d). The CB medium and AM were, therefore, selected to optimize the co-cultured BBB model.

### 2.2. BBB-on-a-Chip Containing Pericytes to Model Barrier Disruption

**Figure 2:**
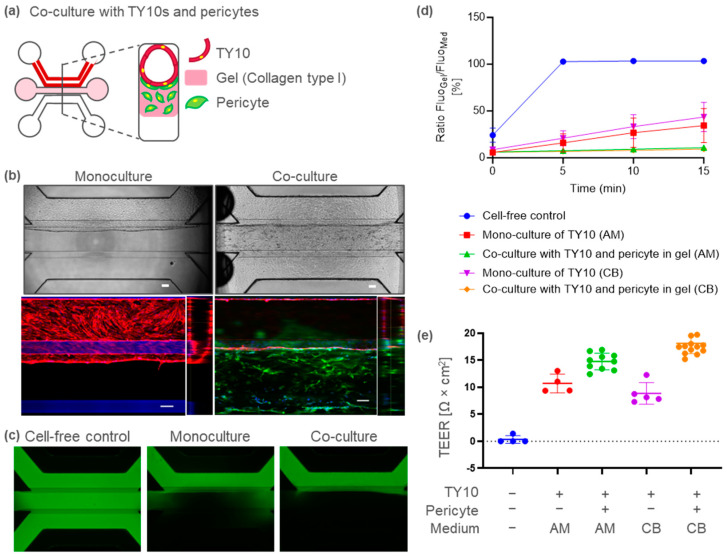
BBB-on-a-chip model containing TY10 brain endothelia and pericytes. (**a**) Schematic representation of the BBB-on-a-chip co-cultured with pericytes. (**b**) Representative phase contrast images of the TY10 monoculture (left, top) and BBB co-culture (right, top) on day 10. Confocal images and cross-section images of a vessel (bottom). A pericyte is shown after PDGFR staining (green) and a TY10 microvessel is shown after CD31 staining (red). TY10 co-cultured with pericytes constructed a tube-like structure. Scale bar is 100 µm. (**c**) Representative fluorescent images of chips perfused with FITC-dextran 20 kDa. Images were acquired on day 10, 15 min after the addition of dye. (**d**) Dextran leakage at different time points for the TY10 monoculture and BBB co-culture models cultured in the ScienCell astrocyte medium (AM) or Cell Biologics endothelium medium (CB). Fluorescent images for quantification were acquired 15 min after adding the dye. (**e**) TEER values of the TY10 mono-culture or BBB co-culture model with pericytes on day 10.

Pericytes play an important role in microvasculature integrity under physiological conditions. To develop a BBB-on-a-chip containing pericytes, pericytes were co-cultured in TY10 microvessels by embedding them in the middle ECM (Figure 2a). Ten days after TY10 and pericyte co-culture, immunostaining for CD31 (endothelia marker) and platelet-derived growth factor receptor (PDGFR; pericyte marker) was performed. In the co-cultured model, microvessels composed of CD31-positive endothelia were formed, as shown in the TY10 monoculture model in Figure 2b. PDGFR-positive pericytes were localized on the basal side, surrounding the TY10 microvasculature (Figure 2b). These structures closely resembled those in the in vivo BBB. 

The BBB-on-a-chip model containing pericytes also exhibited a leak-tight barrier function (Figure 2c,d). The apparent permeability (Papp) was 6–9 × 10^−7^ cm/s in the co-culture as compared to 6–7 × 10^−6^ cm/s in the monoculture model. The TEER was 15 (AM) to 18 (CB) and 9 (CB) to 11 (AM) Ω·cm^2^, respectively, for the co- and monoculture models (Figure 2e). These results suggest that the pericytes functionally supported microvascular barrier integrity in our model.

**Figure 3:**
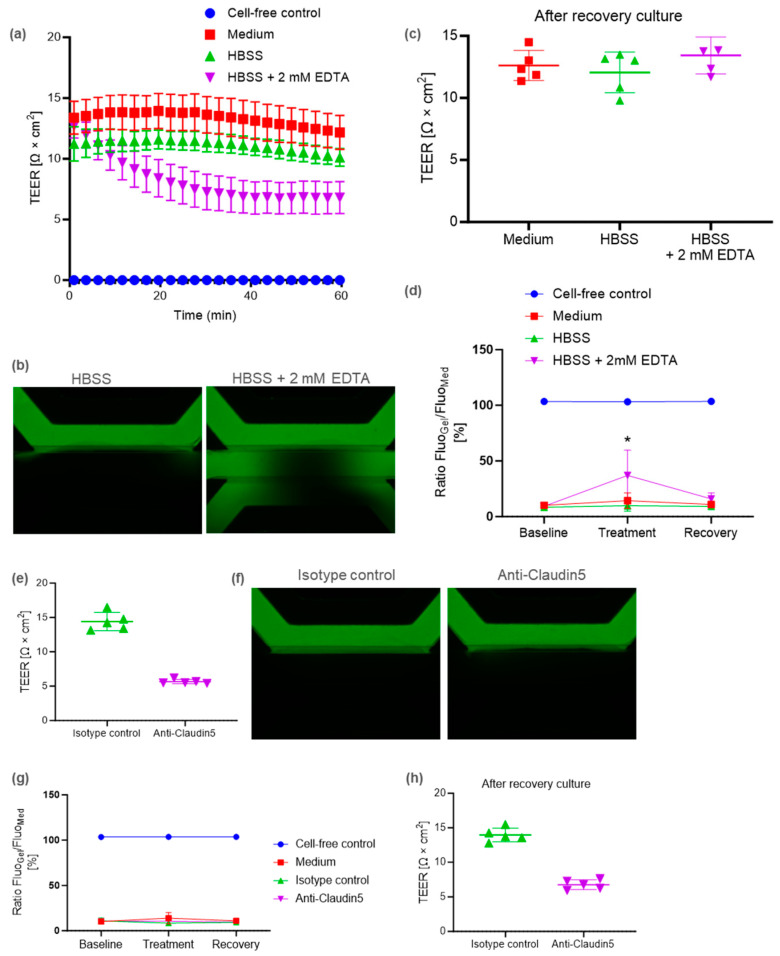
BBB disruption caused by EDTA or anti-CLDN5 rat antibody (rAb) in the BBB-on-a-chip model with pericytes. (**a**) Timelapse of the TEER measurements on day 10. (**b**) Representative fluorescent images of chips perfused with FITC-dextran at 20 kDa after culturing with EDTA or vehicle control (HBSS). (**c**) TEER measurements after the recovery culture. (**d**) Dextran leakage at each time point before (baseline), after culturing with EDTA (treatment), and after the recovery culture (recovery). Data were analyzed using ordinary one-way ANOVA Dunnett’s multiple comparison tests, showing the significant effect of EDTA compared to HBSS (* *p* < 0.05). (**e**) TEER measurements on the BBB cultured with anti-CLDN5 rAb or isotype control antibody. (**f**) Representative fluorescent images of chips perfused with FITC-dextran after culturing with anti-CLDN5 rAb or isotype control antibody. Fluorescent Images were acquired 15 min after adding dye. (**g**) Dextran leakage at each time point before (baseline), after culturing with anti-CLDN5 rAb (treatment), and after the recovery culture (recovery). (**h**) TEER measurements after the recovery culture. Fluorescent images for quantification were acquired 15 min after adding dextran.

Using an established BBB-on-a-chip model with pericytes, we evaluated two BBB-disrupting agents with different mechanisms, i.e., one targeting adherence junctions and the other tight junctions. When the lumen (apical) side was exposed to EDTA, the TEER decreased over time, up to 60 min (Figure 3a). Moreover, dextran leakage was observed after a 1 h exposure to EDTA (Figure 3b). These results suggest that EDTA induced barrier disruption. After the recovery culture in the absence of EDTA, both the TEER and barrier function were restored to the same level as in the vehicle group (Figure 3a,c,d).

CLDN5 is a major tight junction protein in the BBB. CLDN5 neutralizing antibody decreased the TEER in our model (Figure 3e), but it had no effect on dextran leakage at 20 kDa (Figure 3f,g). After the recovery culture, the CLDN5 neutralizing antibody condition continued to induce a decrease in the TEER (Figure 3h), suggesting that it had a long-term effect on brain-barrier integrity.

### 2.3. Tri-Culture BBB-on-a-Chip for Immune Cell Migration

**Figure 4:**
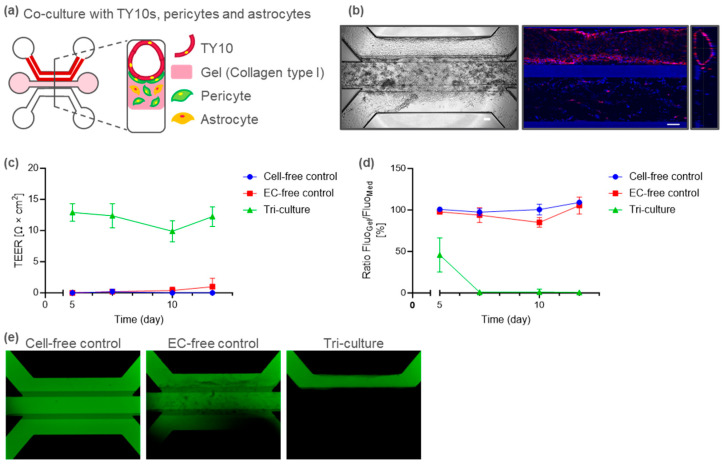
Tri-culture BBB-on-a-chip model. (**a**) Schematic representation of the BBB-on-a-chip model tri-cultured with pericytes and astrocytes. (**b**) Representative images of the BBB tri-culture on day 12. Phase contrast (left). Confocal images and cross-section images of vessel (right). A TY10 microvessel is shown after CD31 staining (red). TY10 tri-cultured with pericytes and astrocytes constructed a tube-like structure. Scale bar is 100 µm. (**c**) TEER values for no cells (cell-free control), no TY10 (EC-free control), and a BBB tri-cultured with pericytes and astrocytes (tri-culture). (**d**) Dextran leakage at different time points for a BBB tri-culture under perfusion. Fluorescent images for quantification were acquired 15 min after adding dye. (**e**) Representative fluorescent images of chips perfused with dextran. Images were acquired on day 12, 15 min after dextran addition.

To establish a tri-culture BBB-on-a-chip model, pericytes and astrocytes were seeded in the middle ECM of the TY10 microvessels (Figure 4a). In the present tri-culture model, TY10 endothelial cells constructed a vessel-like tubule structure under flow, as observed in the mono- and co-culture models (Figure 4b). The tri-culture BBB-on-a-chip model exhibited a detectable TEER after day 5 (Figure 4c) and a stable leak-tight barrier integrity from day 7 to day 12 (Figure 4d). A representative image of the dextran assay on day 12 is shown in Figure 4e.

**Figure 5:**
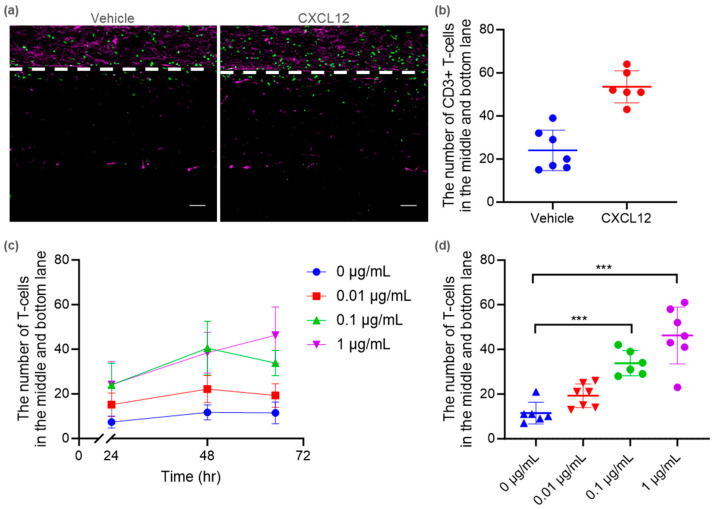
Modeling of transendothelial T cell migration using a tri-culture BBB-on-a-chip model. (**a**) Representative images of transendothelial T cell migration in the tri-culture BBB-on-a-chip model. CD31 and CD3 are visualized as magenta and green, respectively. The white dashed line represents the boundary of the microvessel and ECM. Scale bar is 100 µm. (**b**) Quantification of CD3+ T cell numbers observed outside of the microvessel in the co-culture treated with 0.1 μg/mL CXCL12. (**c**) Quantification of CFSE-labelled T cell numbers observed outside of the microvessel at 24, 48, and 65 h in co-culture treated with CXCL12. (**d**) Quantification of T cell numbers observed outside of the microvessel at 65 h in the co-culture treated with CXCL12. Data were analyzed using ordinary one-way ANOVA Dunnett’s multiple comparison tests, showing the significant effect of CXCL12 (*** *p* < 0.001).

We utilized this tri-culture BBB-on-a-chip model to investigate immune cell migration. Primary human T cells isolated from peripheral blood mononuclear cells (PBMC) were introduced into the TY10 microvessel lumen (top lane). As an endpoint assay, immunostaining was performed to detect CD3+ T cells (Figure 5a). T cell transendothelial migration was observed via gradient C-X-C motif chemokine ligand 12 (CXCL12) (Figure 5b). A live-imaging analysis also demonstrated that the extravasation of carboxyfluorescein succinimidyl ester (CFSE)-labelled T cells increased in response to CXCL12. The number of T cells migrating to the middle and bottom lanes increased in a time-dependent and CXCL12-concentration-dependent manner (Figure 5c). After 65 h, the number of migrated CFSE-labelled T cells significantly increased in response to 0.1 and 1 μg/mL of CXCL12. These observations suggest that the tri-culture BBB-on-a-chip model replicates chemoattractant-mediated transendothelial T cell migration. 

## 3. Discussion

In this study, immortalized human brain endothelial TY10 cells were cultured under flow (Figure 1a). This resulted in the production of a leak-tight 3D microvessel (Figure 1b,c,e). We observed a correlation between the TEER and dextran leakage. TY10 microvessels cultured in the CB medium exhibited TEER values of approx. 10 Ω·cm^2^ and a leak-tight barrier function (Figure 1c,d). In contrast, TY10 microvessels cultured in the EGM-2 medium showed no detectable TEER and a very leaky barrier (Figure 1c,d). To the best of our knowledge, this is the first reported evaluation of the TEER of a microfluidic TY10 model. Given that human BBB cell lines with leak-tight barrier integrity are currently limited [27], this TY10 microvessel model will be useful for functional barrier assays. Since TEER measurements can be performed non-invasively, it will be useful for evaluating the barrier function in multi-sample testing and time-course experiments, such as in drug screening and mechanistic barrier dysfunction investigations.

Several lines of evidence emphasize the importance of pericytes in the development and maintenance of the BBB. PDGF-BB signaling through PDGFR-β is essential for pericyte generation. Mice deficient in either the ligand or receptor completely lack CNS pericytes [33,34]. These mice have increased vascular permeability and die at birth. In addition, pericyte injury is correlated with increased BBB permeability within the hippocampus in elderly patients with mild cognitive impairment [35]. In the present study, PDGFR-positive pericytes were localized on the basal side surrounding the TY10 microvessel (Figure 2b). These structures are very similar to the in vivo BBB. In addition, our co-cultured BBB model, which included pericytes, demonstrated improved barrier integrity (Figure 2c–e). Thus, this model will be useful for pathological research on BBB function in relation to pericytes. 

The TEER value for the Organoplate model using primary human brain endothelial cells was approx. 20 Ω·cm^2^ [21]. In other primary or hiPS-BEC co-culture models, the Papp of 10 kDa dextran was in the range of 1 to 10 × 10^−7^ cm/s [14,29,36,37]. The Papp of 40 kDa was two- to five-fold lower than that of 10 kDa [14,29,36]. Our co-culture model, with a TEER of 15–18 Ω·cm^2^ and a Papp for 20 kDa dextran of 6–9 × 10^−7^ cm/s, appears to have a comparable barrier integrity to other primary or hiPSC-derived BBB models. Since only a limited number of studies have reported both the TEER and dextran Papp, our results may become a valuable benchmark. Various hiPSC models showed a strong leak-tight barrier function with a very high TEER [11,38]. It should be noted that hiPSCs can also differentiate into types of cells other than brain endothelial cells, such as epithelial-like cells [20,26]. To investigate this possibility, it will be useful to evaluate the effect of selective compounds on BBB-specific molecules and to check the expression of epithelial and endothelial markers.

We evaluated two BBB-disrupting agents with different mechanisms, i.e., one targeting adherence junctions and the other tight junctions (TJ). Adherence junctions (AJ) contain transmembrane proteins, known as cadherins, which are responsible for the majority of adhesion between cells [39]. EDTA inhibits the cadherin-mediated cell–cell adhesion junction by capturing metal ions such as Ca^2+^. In our model, EDTA caused a rapid decrease in the TEER (Figure 3a). Furthermore, the removal of EDTA resulted in the recovery of barrier integrity (Figure 3c,d). Ca^2+^ was replenished by medium exchange resulting in AJ recovery. 

CLDN5 is a major TJ constituent protein that is specifically expressed at the BBB. Reduced CLDN5 expression has been reported in patients with psychiatric disorders, including major depression and schizophrenia [40,41]. Hashimoto et al. showed that anti-CLDN5 decreased barrier function in a non-human primate BBB model [42]. However, evidence to demonstrate that CLDN5 inhibition affects the BBB in humans is limited. To the best of our knowledge, for the first time, our results show that CLDN5 inhibition attenuates barrier integrity in a human BBB model, as observed by the decrease in the TEER (Figure 3e). Nitta et al. reported size-sensitive transport by CLDN5 in the BBB [43]. In claudin-5-deficient mice, the brain penetration of small molecules (<800 Da) increased, but that of 10 kDa dextran was not altered [43]. In the present study, anti-CLDN5 reduced the TEER but had no effect on the leakage of 20 kDa dextran (Figure 3e–g). These results are consistent with an in vivo study using Cldn-5-deficient mice [43]. In addition, our results also imply that targets other than CLDN5 may be involved in the intercellular transport of macromolecules. Our pericyte co-culture model suggests that the AJ is important for the intercellular transport of macromolecules across the BBB. In our study, the TEER was still reduced one day after medium exchange (Figure 3h). It has been reported that the CLDN5 neutralizing antibody used in our study binds to CLDN5 on the cell membrane, and then internalizes within the cell [42]. We speculate that CLDN5 internalization did not recover sufficiently within the recovery culture for 24 h. The present study demonstrates that it is feasible to modulate the BBB using an antibody-based approach. We expect our human BBB-on-a-chip model to be utilized in experiments exploring the biological properties of the BBB in humans, in CNS delivery research using BBB modification, and in pathophysiological investigations of autoimmune encephalitis caused by autoantibodies.

Multiple lines of evidence indicate that T cells play a central role in both mediating and regulating the pathophysiology of multiple sclerosis (MS) [44,45]. Autoreactive T cells migrate across the BBB and cause damage to central neurons and their myelin sheaths. The histological analysis of MS lesions has revealed that CD8+ T cells are often localized adjacent to regions of demyelination [46]. Several studies have reported that CXCL12 levels are elevated on astrocytes in active MS lesions [47,48]. Thus, CXCL12 may be an attractive target for the treatment of MS. In addition, Parkinson’s disease (PD) is a neurodegenerative disorder characterized by the loss of dopamine-containing neurons in the substantia nigra (SN). Post-mortem SN specimens from patients with PD show significant invasion of both CD8+ and CD4+ T cells [49]. In Lewy body dementia (LBD), T cells adjacent to Lewy bodies and neurons have been observed in post-mortem LBD brains [50]. Gate et al. identified the upregulation of CXCR4, a receptor of CXCL12, in CD4+ T cells in the CSF of LBD patients [50], suggesting the CXCR4–CXCL12 signaling axis as a potential therapeutic target for LBD. To the best of our knowledge, the present study is the first to model transendothelial T cell migration using a microfluidic tri-culture human BBB model that includes brain endothelia, pericytes, and astrocytes (Figure 5). Our model was developed using scalable cells and is applicable to routine use in drug discovery research. In a past study, primary brain endothelial cells were exposed to TNFα for 24 h to induce an inflammatory state such as that induced by the upregulation of intercellular adhesion molecule 1 on brain endothelia. Subsequently, a T cell migration assay was conducted [21]. In our tri-culture model with astrocytes and pericytes, we detected transendothelial T cell migration without pre-treatment with TNFα. This represents obvious progress in terms of model complexity compared with monoculture-based BBB models. As crosstalk between T cells and astrocytes during neuroinflammation has been reported [51], our model represents a powerful tool for investigating the role of astrocytes in inflammation and their interaction with endothelia and immune cells. Accordingly, our human BBB model will be useful for clinically analyzing the mechanism of immune cell infiltration. Further applications are also expected, such as elucidating immune cell migration in pathological conditions using patient-derived blood and cerebrospinal fluid samples and evaluating the effect of CAR-T on brain tumors. In addition, it can be expanded to introduce other cells (such as nerve cells), depending on the application.

Unlike the Transwell system, our model has a parallel structure that allows the movement of substances to be visualized. The immunostaining of T cell and endothelial markers (CD3 and CD31) demonstrated the transendothelial migration of human T cells in response to CXCL12 (Figure 5a,b). In addition, the live-imaging analysis successfully detected the dynamic migration of T cells occurring in a time-dependent and CXCL12-concentration-dependent manner (Figure 5c,d). These results suggest that our BBB-on-a-chip model can provide substantial information on the three-dimensional spatial distribution and kinetics of cells of interest in disease states. These data will be valuable for the mechanistic understanding of various factors in neuroinflammatory disease.

Our model has certain limitations. For example, our TEER values were lower than the in vivo values previously measured in rodents [52,53]. It should be noted that the TEER has not been measured in humans. In addition, flow in our model is bidirectional, unlike the case in vivo. Considering these limitations, a comparison of in vitro TEER values with those in vivo may be challenging. 

In conclusion, we established a novel human BBB-on-a-chip for the modeling of barrier dysfunction and immune cell migration for drug discovery research. Our model replicates several morphological features of the in vivo BBB, including (1) a microvessel structure consisting of TY10 brain endothelial cells, (2) pericytes/astrocytes localized on the basal side surrounding brain microvessels, and (3) fluid flow on the apical side of the BBB. For the first time, we characterized the barrier integrity of a mono-/co-/tri-culture TY10 microfluidic model using TEER measurements and a dextran assay, demonstrating transendothelial T cell migration using scalable cells, namely, TY10, pericytes, and astrocytes. The BBB chip showed improved barrier integrity under co-culture with pericytes, and reproduced transendothelial T cell migration under flow, as reported in MS and PD patients [44,45,49]. Barrier dysfunction was also successfully modeled by two BBB-disrupting agents based on different mechanisms, i.e., one targeting adherence junctions, the other tight junctions. Our model was also characterized using anti-CLDN5, which is selective for a BBB-specific molecule, indicating its potential use for the pathophysiological investigation of BBB disruption caused by autoantibodies. In addition, our study suggests that CLDN5 inhibition attenuates barrier integrity in a human BBB-on-a-chip model. The “fit-for-purpose” approach has been proposed for the use of human models depending on the context of use (CoU) in drug discovery research and development [54]. Currently, there are no promising therapeutic agents for CNS diseases that target the BBB; thus, a significant unmet need exists. Due to species differences, animal models do not necessarily reflect human biology, and the establishment of a human disease model is highly attractive. Indeed, the FDA recently adopted legislation that allows the utilization of microphysiological systems, such as organ-on-a-chip models in drug discovery research and development, as an alternative to animal testing [55]. The platform established in this study has potential applications in a wide range of fields related to the BBB, including drug discovery research and screening, and studies elucidating the mechanisms of pathological conditions.

## 4. Materials and Methods

### 4.1. Cell Culture

Cell lines of immortalized human brain microvascular endothelial cells (TY10) [30,31], immortalized human brain pericytes (hBPCT) [56], and immortalized human astrocytes (hAST) [57] were provided by Yamaguchi University, Japan. Cells were cultured at 33 °C, 5% CO_2_ to allow optimal cell expansion in a collagen type I-coated 60 mm dish (356401, Corning, NY, USA) or 100 mm dish (4020-010, Iwaki, AGC Techno Glass Co. Ltd., Yoshida, Japan). Cell lines were immortalized with an hTERT and a temperature-sensitive SV40 large-T antigen, which allowed the cells to grow at the permissive temperature of 33 °C. TY10 cells were cultured in EGM-2 (CC-3162, LONZA, Basel, Switzerland), which was supplemented with 20% fetal bovine serum (FBS). hBPCT cells were cultured in pericyte medium (#1201, ScienCell, Carlsbad, CA, USA). hAST cells were cultured in astrocyte medium (AM, #1801, ScienCell). 

### 4.2. Culture of TY10 Microvessels in OrganoPlate 3-Lane

The OrganoPlate 3-lane platform (4004-400B, MIMETAS B.V., Oegstgeest, The Netherlands) was used for all experiments. To establish a BBB model via the monoculture of TY10, a collagen-I gel was dispensed in the gel inlet, filling the middle channel. TY10 cells were seeded and cultured in the top channel as previously described [12]. In short, a TY10 cell suspension of 1.5 × 10^7^ cells/mL was prepared and 2 µL was seeded in the inlet of the top channel. Fifty microliters of medium was added to the top channel inlet and the plate was incubated on its side for 1 h in the incubator to allow the cells to sediment against the collagen-I gel and attach. After incubation, 50 µL of medium was added to the top channel outlet. The OrganoPlate was placed on an interval rocker (Mimetas Rocker Mini, MIMETAS B.V. Oegstgeest, The Netherlands) switching between a + 7° and − 7° inclination every 8 min, allowing bidirectional flow. Cells were cultured at 33 °C and 5% CO_2_ to allow full cell coverage of the ECM gel. During the entire culture period, only the top channel, which contained the TY10 microvessel, was perfused with culture media to allow the optimal endothelial barrier strength. The medium was refreshed every 2–3 days. The following media were used to assess their influence on barrier function: Cell Biologics Complete Human Endothelial Cell Medium (CB medium, #H1168, Cell Biologics, Chicago, IL, USA), EGM-2, and AM.

### 4.3. BBB-on-a-Chip Co-/Tri-Cultured in OrganoPlate 3-Lane

To co-culture with TY10 and hBPCT as a BBB co-culture model, hBPCT cells were embedded into a collagen-I solution as previously described. In short, hBPCT cells were counted and 2.5 × 10^6^ cells/mL was resuspended in collagen I. An hBPCT cell suspension in collagen I (2 µL) was loaded into the gel inlet, filling the middle channel. After collagen-I gelation, TY10 cells were seeded and cultured in the top channel, as described in the previous section. The CB medium and AM were used to assess their influence on barrier function.

To tri-culture with TY10, 5.0 × 10^6^ cells/mL hBPCT cells and 7.5 × 10^6^ cells/mL hAST cells were embedded into a collagen-I solution and cultured in the middle channel. TY10 cells were seeded in the top channel in the same way as previously described. TY10 cells were cultured in the CB medium for 3 days, then AM was used to culture them. 

### 4.4. TEER Measurements

The TEER was measured at different time points using an automated multichannel impedance spectrometer designed for use with the OrganoPlate (OrganoTEER, MIMETAS B.V.). Prior to all experiments, the OrganoTEER electrode boards were disinfected by spraying the electrodes’ surface with 70% ethanol and allowing them to dry in a sterile flow cabinet. Before each measurement, all inlet and outlet media were refreshed and the OrganoPlate was placed at room temperature to equilibrate for 30 min. A single electrode board and measurement unit were assembled and inserted into the OrganoPlate. Then, the TEER value was measured at room temperature. The electrode board of the OrganoTEER was matched to the OrganoPlate, such that when the OrganoPlate was placed in the OrganoTEER, electrode pairs were inserted in the medium in all inlet and outlet wells, connecting to the basal and apical sides of all the tubes. In the case of the continuous long-term TEER value measurement, the OrganoPlate with a TEER measurement unit was placed on a MIMETAS Rocker Mini to maintain a fluidic culture stimulation in the incubator (33 °C, 5% CO_2_). Data were analyzed using the OrganoTEER software (ver. 2.1.0) and the TEER value was output as Ohm × cm^2^ by multiplying by the tubule–ECM interface. 

### 4.5. Barrier Integrity Assay

Chips were washed with culture medium (50 µL on all inlets and outlets, 1 × 5 min) to ensure proper flow profiles during the subsequent barrier integrity assay. Next, all media were aspirated from the chips and 20 µL of the medium without a fluorescent compound was added to the basal side of the chips (the bottom channel inlets and outlets). Medium containing 0.5 mg/mL FITC-dextran (20 kDa, FD20S, Sigma, St. Louis, MO, USA) was added to the top channel, which contained the TY10 microvessel (40 µL on inlet; 30 µL on outlet) and image acquisition was started. Fluorescent molecule leakage from the lumen of the microvessel into the adjacent middle channel was automatically imaged using a BZ-X800 (KEYENCE, Osaka, Japan). The fluorescent signal ratio in the top channel vs. the middle channel (Ratio FluoGel/FluoMed) was determined. The Papp was determined using the curve fitting approach, as described elsewhere [58].

### 4.6. Barrier Disruption Assay

For barrier disruption experiments, the BBB-on-a-chip co-cultured with TY10 and pericytes was cultured for 10 days. As barrier disruption factors, EDTA solution (06894-14, NACALAI TESQUE, INC., Kyoto, Japan) and anti-CLDN5 rat monoclonal antibody (Anti-Claudin5, 014-28101, FUJIFILM Wako Pure Chemical Corporation, Osaka, Japan) were used. IgG2b rat-mono Isotype Control (MAB0061, Clone#141945, R&D systems, Bio-techne Corporation, Minneapolis, MN, USA) was used as the isotype control for anti-CLDN5.

Prior to exposure, TEER measurements and a barrier integrity (BI) assay were performed on all chips. After the measurements, the medium was refreshed and OrganoPlate was placed on a MIMETAS Rocker Mini in the incubator (33 °C, 5% CO_2_) for over 20 min to allow the OrganoPlate temperature to equilibrate. 

For the barrier disruption assay using EDTA, 2 mM of EDTA solution was prepared via dilution with HBSS (14025092, Gibco, Thermo Fisher Scientific, Waltham, MA, USA). Next, the medium was aspirated from the chips, after which 50 µL of 2 mM EDTA, HBSS, or CB medium was added to the inlet and outlet. To minimize temperature fluctuation, the OrganoTEER assembly was placed on the MIMETAS Rocker Mini in the incubator (33 °C, 5% CO_2_). The first TEER measurement was taken immediately after the medium change. TEER measurements were taken every subsequent 2 min and 40 s for approximately 1 h (total 23 times). After the completion of the TEER timelapse, the OrganoPlate was removed from the TEER device and a BI assay were performed. As a recovery culture, all media or solutions were substituted with fresh CB medium and chips were cultured for 2 days.

For the barrier disruption assay using anti-CLDN5, 150 µg/mL of anti-CLDN5 or isotype control antibody was prepared via dilution with CB medium. The medium was aspirated from the chips, after which 30 µL of the medium containing the antibody was added to the top channel inlet and outlet. The OrganoPlate was placed on the MIMETAS Rocker Mini in the incubator (33 °C, 5% CO_2_). During the entire culture period, only the top channel, which contained the TY10 microvessel, was perfused with culture media. After 12.5 h, the TEER value was measured and a BI assay was performed. As a recovery culture, all media were substituted with fresh AM and chips were cultured for 1 day.

After the recovery culture, TEER measurements and a BI assay were performed on all chips.

### 4.7. T Cell Isolation, Stimulation, and Labelling

T cells were isolated from Cryopreserved Human PBMC (4W-270, LONZA, Basel, Switzerland). PBMCs were thawed and washed three times with RPMI1640 (R8758, Sigma) supplemented with 10% FBS, 1% L-glutamine (G7513, Sigma), 1% penicillin-streptomycin (15070063, Gibco, Sigma), and 20 U/mL DNase (D4513-1VL, Sigma). T cells were isolated from PBMC using a Pan T cell isolation kit (130-096-535, Miltenyi Biotec, Bergisch Gladbach, Germany) following the corresponding protocol. 

To stimulate T cells, isolated T cells were suspended in RPMI1640 supplemented with 10% FBS, 1% L-glutamine, 1% penicillin-streptomycin, 50 U/mL IL-2 (200-02, PeproTech, Thermo Fisher Scientific), and CD3/CD28 Human T-activator Dynabeads (11131D, Gibco, Thermo Fisher Scientific) at a bead-to-T-cell ratio of 1:1. These T cells were seeded in 24-well plates (3820-024, Iwaki, AGC Techno Glass Co. Ltd., Yoshida, Japan) and cultured for 4 days in the incubator (37 °C, 5% CO_2_).

Prior to adding the T cells to the endothelial tubules in the OrganoPlate, stimulated T cells were labelled using Cellstain CFSE (341-07401, Dojindo, Kumamoto, Japan). A total of 5 mM of CFSE stock in dimethylsulfoxide (DMSO) was diluted to a working concentration of 5 µM in EGM-2. T cells were harvested and pelleted (300× *g*, 5 min) before being resuspended in an appropriate volume of CFSE working solution to achieve a final concentration of 1.0 × 10^7^ cells/mL. Cells were then placed in the incubator (37 °C, 5% CO_2_). After 15 min, the same volume of EGM-2 was added and cultured for an additional 5 min. After the incubation period, cells were pelleted (300× *g*, 5 min) and resuspended with 2 mL of RPMI1640 supplemented with 10% FBS, 1% L-glutamine, and 1% penicillin-streptomycin. Cells were counted and pelleted (300× *g*, 5 min) again. Then, the appropriate volume of the above medium was added to the T cells to achieve a final concentration of 4 × 10^5^ cells/mL.

### 4.8. Immune Cell Migration Assay

To investigate immune cell migration, the BBB-on-a-chip tri-cultured with TY10, pericytes, and astrocytes was cultured for 7 days. The culture medium of all channels, which contained the TY10 microvessel, was aspirated and 50 µL of T cell suspension containing 20,000 T cells was added to the top channel inlet. Thereafter, 50 µL of RPMI1640, supplemented with 10% FBS, 1% L-glutamine, and 1% penicillin-streptomycin, was added. A total of 50 µL of either plain RPMI1640 medium, the aforementioned medium, or a medium containing 0.01, 0.1, 1 µg/mL CXCL12 (350-NS-050/CF, R&D systems, Bio-techne Corporation) was added to the bottom perfusion channel inlet and outlet. Then, the OrganoPlate was incubated (33 °C, 5% CO_2_) on the MIMETAS Rocker Mini for 65 h. The T cells were imaged at 24, 48, 65 h using an IN Cell Analyzer 6000 (GE HealthCare, Chicago, IL, USA). Images were acquired at a 10× magnification at 5 µm increments along the height of the microfluidic channel. The analysis was based on max intensity projection images of 37 z-slices. After co-culture with T cells, the plates were fixed in 4% paraformaldehyde phosphate buffer solution (163-20145, FUJIFILM Wako Pure Chemical Corporation, Osaka, Japan) and stored at 4 °C. The quantification of the T cell dynamics was performed using the High-Content Analysis Software CellPathfinder (ver.3.05.02.02, YOKOGAWA, Musashino, Japan). Before image analysis, all images were converted using CellPathfinderMetaDataCreator (ver.1.01.04.01, YOKOGAWA, Musashino, Japan). 

### 4.9. Immunocytochemistry

After fixation, cultures in the OrganoPlate were permeabilized with 0.3% Triton X-100 (T8787, Sigma) in DPBS (045-29795, FUJIFILM Wako Pure Chemical Corporation). Cells were incubated with blocking solution (2% FBS, 2% bovine serum albumin (A9576, Sigma) and 0.1% Tween-20 (P9416, Sigma) in DPBS) for 45 min. Primary antibody was incubated for 2 h on the MIMETAS Rocker Mini (switching between a + 5° and −5° inclination every 2 min), after which the secondary antibody was incubated for 30 min. Washing was performed by replacing solutions with a washing solution (4% FBS in DPBS), after which plates were incubated for 5 min. This step was performed 1–3 times after each step. The following antibodies were used: anti-Human CD31 (M0823, Dako, Agilent, CA, USA), anti-CD3 (ab5690, Abcam, Cambridge, UK), goat anti-rabbit Alexa Fluor 488 (A11034, Thermo Fisher), and goat anti-mouse Alexa Fluor 647 (A21236, Thermo Fisher). Nuclei were stained using NucBlue Live ReadyProbes Reagent (R37605, Thermo Fisher). All steps were performed at room temperature. Cells were imaged with the CellVoyager CQ1 Benchtop High-Content Analysis System (YOKOGAWA). 

### 4.10. Statistical Analysis

Means of three or more groups were assessed using the one-way ANOVA test for statistically significant differences. Multiple comparisons were accounted for using Dunnett’s tests. Graphs were plotted and statistical analyses were performed using GraphPad Prism (GraphPad Software, ver.10.1.2, San Diego, CA, USA). Data points in Figures represent individual chips. Error bars in Figures show the standard deviation of the mean.

## Data Availability

Data are contained within the article.
